# Indium Tin Oxide Nanowire Arrays as a Saturable Absorber for Mid-Infrared Er:Ca_0.8_Sr_0.2_F_2_ Laser

**DOI:** 10.3390/nano12030454

**Published:** 2022-01-28

**Authors:** Yuanhao Zhao, Mengyu Zong, Jie Zheng, Zhen Zhang, Qianqian Peng, Shouzhen Jiang, Jie Liu, Jingjing Liu, Liangbi Su

**Affiliations:** 1 Shandong Provincial Engineering and Technical Center of Light Manipulations and Shandong Provincial Key Laboratory of Optics and Photonic Device, School of Physics and Electronics, Shandong Normal University, Jinan 250358, China; zyhsdnu2021@163.com (Y.Z.); zmy_6622@163.com (M.Z.); sdnuzhengjie@163.com (J.Z.); pengqq@sdnu.edu.cn (Q.P.); jiang_sz@126.com (S.J.); 2 CAS Key Laboratory of Transparent and Opto-Functional Inorganic Materials, Synthetic Single Crystal Research Center (SSCRC), Shanghai Institute of Ceramics, Chinese Academy of Sciences, Shanghai 201899, China; catewolf@outlook.com; 3 State Key Laboratory of High Performance Ceramics and Superfine Microstructure, Shanghai Institute of Ceramics, Chinese Academy of Sciences, Shanghai 201899, China

**Keywords:** mid-infrared laser, saturable absorber, passively Q-switched, nanowire arrays

## Abstract

We demonstrated a passively Q-switched Er:Ca_0.8_Sr_0.2_F_2_ laser with indium tin oxide nanowire arrays as an optical modulator in the mid-infrared region. In the Q-switched regime, the maximum output power of 58 mW with a slope efficiency of 18.3% was acquired. Meanwhile, the minimum pulse duration and highest repetition rate of the stable pulse trains were 490 ns and 17.09 kHz, corresponding to single pulse energy of 3.4 μJ and peak power of 6.93 W, respectively. To the best of our knowledge it was the first time that indium tin oxide nanowire arrays were employed as a saturable absorber to make pulse lasers carried out at 2.8 μm. The experimental data show that indium tin oxide nanowire arrays can be employed as a competitive candidate for saturable absorber in the field of mid-infrared solid-state lasers.

## 1. Introduction

Recently, mid-infrared (MIR) lasers have aroused great interest contributing to a wide range of applications in multifarious aspects, such as strong field physics, atmospheric environmental monitoring, biomedical, communication, military, industry and so forth [1,2,3]. A laser at 3 μm is located around the strong absorption peak of H_2_O, which was utilized as ideal laser source for high precision laser surgery [4]. Generally speaking, one common method to acquire MIR lasers is a specific technique called nonlinear frequency transformation by optical parametric oscillator [5,6]. Yet, the preceding laser system is not only limited by the size and damage threshold of nonlinear crystals, but is also hard to control due to the existence of phase mismatch and high-order dispersion [7].

**Figure 1:**
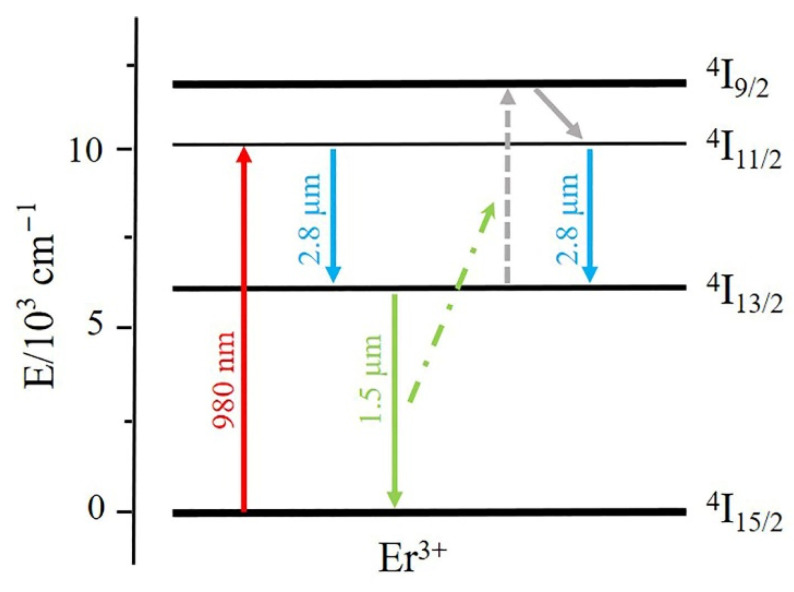
Energy level configuration diagram of Er^3+^ ions.

In the MIR region, Er-doped crystals are known as great gain materials. On the one hand, according to the energy level of Er^3+^ ions, transition from the ^4^I_11/2_ state to ^4^I_13/2_ state can directly emit laser at 2.8 μm, as illustrated in Figure 1. On the other hand, calcium fluoride (CaF_2_) crystals are well-known with their representative fluorite structure and strontium fluoride (SrF_2_) crystals possess similar structure. CaF_2_, a defect system, has superior properties that trivalent rare-earth dopants possess aggregation phenomena. Dopants can be contained in its structure while the structural integrity will not be destroyed [8]. The excellent fluoride structure can prompt trivalent ions into clusters even at low doping concentration of Er^3+^. Therefore, the spacing between ions is shortened and the energy up-conversion process can be largely enhanced to some extent. Besides, fluoride crystals possess lower phonon energy (CaF_2_: 322 cm^−1^, SrF_2_: 280 cm^−1^) compared with oxide substrates (e.g., YAG: 700 cm^−1^, Y_2_O_3_: 591 cm^−1^), which can reduce the possibility of nonradiative transition [9,10,11,12,13,14]. Thus, the self-terminating effect of Er^3+^ is suppressed and researches in recent years have been based on Er-doped CaF_2_ or SrF_2_ crystals to obtain mid-infrared lasers. In 2019, an Er^3+^:Ca_0.8_Sr_0.2_F_2_ mixed crystal laser with a 1.41 W CW output power was achieved by Liu Jingjing [15] and in 2020, Zong et al. firstly demonstrated the laser performance of Er:CaF_2_ single-crystal fiber [16].

Q-switched technology is an effective method to obtain a pulse laser with narrow pulse duration (μs and ns magnitude) and large energy. It mainly includes active Q-switching and passive Q-switching. The active devices in the 3 μm band (e.g., acousto-optic crystal, electro-optical crystal) possess higher loss and are more expensive compared with passive Q-switched components. In practice, the generation approaches of passively Q-switching are largely on the basis of an efficient optical modulator. Still, mature commercial saturable absorbers (SAs) in the MIR region have yet to be developed. Therefore, SAs with low cost, easy preparation and great stability should be explored and manufactured in the MIR region. With the emergence of low-dimensional materials at present [17,18,19], manifold novel low-dimensional materials are considered as competitive candidates among SAs. Low-dimensional nanoscale materials, such as transition metal dichalcogenides (TMDs) [20,21], black phosphorus (BP), MXene [22,23] and perovskite materials, were reported.

We specifically display the nonlinear saturable absorption properties of one characteristic low-dimensional material, indium tin oxide nanowire arrays (ITO-NWAs) at 2.8 μm. ITO belongs to one of the degenerate semiconductor transparent conducting oxides, and possesses great optical nonlinear properties, high damage threshold and high modulation speed [24,25]. The spectral region, in which the real component of dielectric permittivity nears zero, is termed as an epsilon-near-zero (ɛ~0, ENZ) region, exhibiting infinite phase velocity of propagating light in material, large optical nonlinearity and near-zero refractive index [26,27,28]. In the ENZ region of ITO, it has all the advantages above, such as distinct optical nonlinear response, ultra-large intensity-dependent refractive index with an extremely fast recovery time of 360 femtoseconds and low linear optical losses [29,30]. In 2003, Wang et al. first successfully observed ITO in the form of nanowires and ITO nanowires demonstrating outstanding performance among which the most prominent ones include high transparency in the visible region (90%) and conductivity properties [31,32,33]. All of the above characteristics have led to ITO being exhaustively researched, analyzed and used in optoelectronics recently. By now, several reports have treated ITO as a SA and inserted ITO into fiber lasers to realize Q-switched lasers or mode-locked lasers. Almost all lasers are emitted around the 1500 nm band [34,35,36,37,38,39,40,41,42]. In 2020, Feng et al. firstly reported that ITO-NWAs were employed as a SA in all-solid-state lasers and they realized passively Q-switched pulse lasers at 1.0, 1.3 and 2.0 μm [43]. Up to now, there are few related researches around 3 μm.

In this paper, the nonlinear saturable absorption properties of ITO-NWAs at 2.8 μm were vividly characterized. To the best of our knowledge, using ITO-NWAs to perform as a SA and being inserted into an optical resonator, a passively Q-switched laser around 2.8 μm was realized for the first time. While the maximum absorbed pump power got up to 2.38 W, a stable pulse laser was obtained with 17.09 kHz repetition rate and 490 ns minimum pulse duration, corresponding to 3.4 μJ single pulse energy and 6.93 W peak power. We experimentally indicated that ITO-NWAs could perform well as a SA not only in fiber lasers, but also in all-solid-state pulse lasers. In the MIR region, ITO-NWAs are promising to become competitive candidates for an optical SA in the ultrafast pulse laser region. 

## 2. Materials and Methods

**Figure 2:**
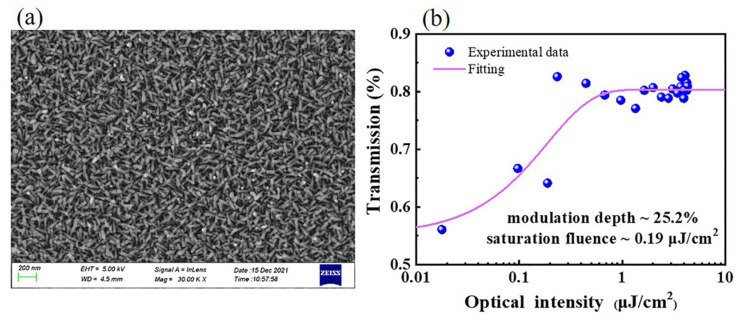
Characterizations of ITO-NWAs surface topography and nonlinear transmission: (**a**) the SEM image at a 200 nm scale, (**b**) the nonlinear transmission at 2.8 μm.

ITO-NWAs, a unique form of ITO nanostructures, were successfully obtained through chemical vapor deposition (CVD) method. Furthermore, 0.004 g of indium oxide, 0.036 g of tin oxide and equal mass of graphite powder were all merged together. Then, on a mica sheet (10 × 10 × 1 mm^3^), the final product of ITO-NWAs was catalyzed by Au nanoparticles and obtained with success by double temperature zone DT-PECVD [43]. Figure 2a shows the surface topography of the particles which were analyzed in a 200 nm scale bar by scanning electron microscopy (SEM) (ZEISS, Sigma 500, Oberkochen, Germany). The SEM image indicated that all the ITO nanowires are uniformly distributed and formed rows of neat nanowire arrays.

SAs can be regarded as two-level systems whose absorption coefficients at central frequency can be expressed by the following equation:

(1) β=β01+IIs′

where β^0^ is the pump light absorption rate of SA under small signal, I is the pump light intensity in the SA and I^′^_s_ is the saturation intensity. The absorption coefficient β decreases with the increase of incident pump power. When the incident pump power is large enough, the β is about 0. 

After ITO was inserted into the resonant as Q-switch, the β of ITO was at a high level due to the weak incident pump power at the initial stage, resulting in a large energy loss in the cavity and the laser could not be generated by oscillation amplification. As the laser gain medium particles accumulated in the upper level and the spontaneous radiation enhanced gradually, laser intensity in the cavity increased and the β decreased when the ratio of I/I^′^_s_ could no longer be ignored. While the gain of the cavity was equal to the loss, the laser started oscillating, resulting in an avalanche of stimulated radiation. In this experiment, the detailed characterization of ITO-NWAs of the nonlinear saturable absorption properties at 2.8 μm are shown in Figure 2b. All quantitative data were drawn as dots and the nonlinear fitting curve was accessed via the following formula:

(2) $$T\left(I\right)=1-\Delta T\times \exp\left(\frac{-I}{I_{\mathrm{sat}}}\right)-T_{\mathrm{ns}}$$

where I is the incident fluence, ΔT is modulation depth and I_sat_ and T_ns_ are correspondingly the saturable intensity and nonsaturable absorbance of the ITO-NWAs. A 2.8 μm pulse laser (repetition rate: 100 kHz, pulse width: 220 fs) was employed as a laser source to examine nonlinear absorbed properties. The transmittance of ITO-NWAs had an escalating trend while the input power was augmented until ITO-NWAs were saturated, demonstrating the great nonlinear saturable absorption performance of ITO-NWAs at 2.8 μm. As shown in Figure 2b, the smooth fitting curve of the data indicated that the saturable fluence was 0.19 μJ/cm^2^ and the modulation depth was 25.2% at 2.8 μm.

## 3. Experiment and Discussion

To investigate the optical performance of ITO-NWAs at 2.8 μm, a compact Er:Ca_0.8_Sr_0.2_F_2_ laser was established. A 3 × 3 × 10 mm^3^ 3 at.% Er:Ca_0.8_Sr_0.2_F_2_ crystal, which was wrapped up with thin indium foil and embedded into the copper billet with water cooling installed to increase heat dissipation, was placed in a V-type cavity. M_1_, a plane mirror, was employed as input mirror which was anti-reflectively coated (ARC) for 974 nm and high-reflectively coated (HRC) for 2.9 μm. M_2_, a concave mirror with curvature radius of 100 mm was ARC for 974 nm and HRC for 2.9 μm, respectively. M_3_, partial- reflectively coated for different transmissions (T = 1%, 2%, 3%, 5%) in 2.7–2.95 μm on both sides, were used as output coupling mirror (OC). The pumping source, a commercial CW laser diode (LD) emitted at 976 nm which was coupled by fiber (numerical aperture of 0.22, fiber core size of 105 μm). The pump laser was focused at the middle of the crystal by a coupling system (compression ratio of 1:2), which meant the spot radius on the laser crystal was about 105 μm. By recording the pump power measured in front of and behind the gain medium, respectively, the absorption efficiency of the Er:Ca_0.8_Sr_0.2_F_2_ crystal was calculated to be 71%.

**Figure 3:**
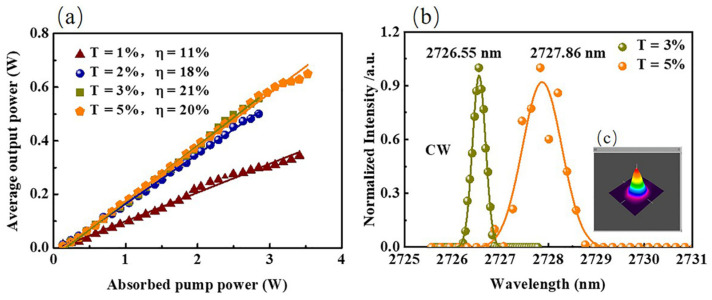
(**a**) Average output power versus the absorbed pump power for CW lasers at 2.8 μm; (**b**) the spectra for the 2.8 μm CW lasers; inset (**c**) shows 3D light intensity distribution.

The CW V-type laser was carried out in the aforementioned optical resonator system without containing ITO-NWAs as SA. The CW laser at 2.8 μm was achieved with different OCs, while the L_1_ was about 81 mm and the L_2_ was about 114 mm, calculated by propagation ABCD matrix theory. The performances of the CW laser, measured by power meter (30A-SH-V1, Israel), are demonstrated as shown in Figure 3a. The lasing thresholds of CW lasers were 131 mW and by continually increasing the incident power, the average output power rose accordingly. With the OC of T = 3%, 558 mW average output power was delivered when the absorbed pump power of 2.8 W. When we used the suitable OC, the average output power could be greatly improved. By exchanging the transmittance of the OC, we acquired a higher output power of 650 mW with the OC of T = 5% under the absorbed pump power of 3.5 W. The spectra of the CW laser are demonstrated with the OC of T = 3% and T = 5% separately in Figure 3b. Meanwhile, the intensity distribution of 3D light was recorded (NS2-Pyro/9/5-PRO, Photon), shown in Figure 3c. The image implied that the CW laser was in TEM_00_ mode. The peaks of the emission spectra were located at 2726.55 nm with full width at half maximum (FWHM) of 0.32 nm with the OC of 3% and 2727.86 nm with FWHM of 0.97 nm with the OC of 5%.

When transmittance of OC was 1% and 2%, the relevant output power of CW laser apparently showed lower performance compared with 3% and 5%. In the following Q-switched experiment, only the results with OC of T = 3% and 5% will be further explored and discussed. 

**Figure 4:**
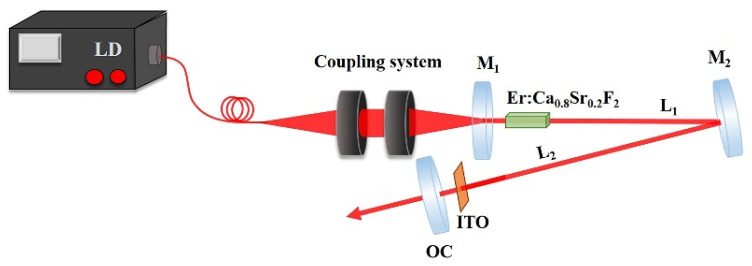
Experimental configurations for Q-switched laser.

The experimental configuration of the Q-switched laser was shown in Figure 4. Through bedding ITO-NWAs into the optical resonator as a SA at optimum location with a three-dimensional adjustment frame and augmenting the incident pump power, a stable pulse laser was obtained. The trains of pulse laser were visualized by the photodetector with rapid response speed (VIGO System S.A. with a rising time of about 3.5 ns) and the digital oscilloscope (Tektronix DPO4104, 1 GHz bandwidth, 5 G samples/s). 

**Figure 5:**
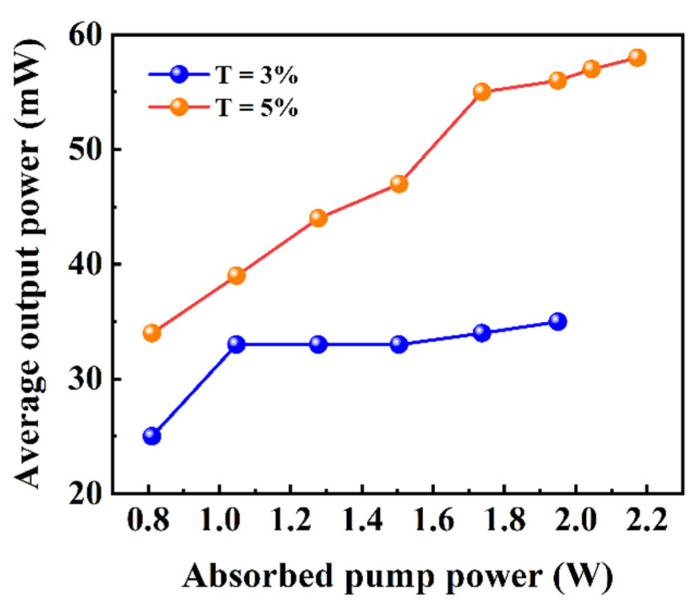
Average output power when T = 3% and 5% of the Q-switched lasers at 2.8 μm.

**Figure 6:**
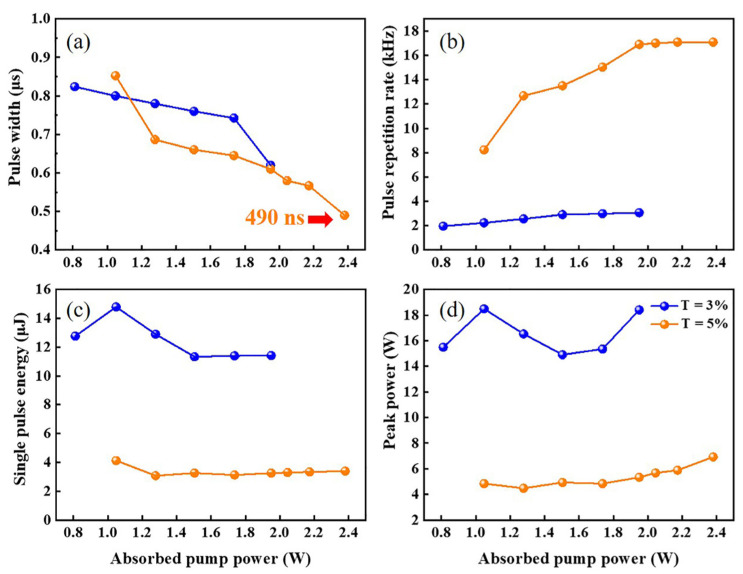
Pulse width (**a**), pulse repetition rate (**b**), single pulse energy (**c**) and peak power (**d**) when T = 3% and 5% of the Q-switched lasers at 2.8 μm.

When the absorbed pump power of the crystal reached 811 mW, stable pulse trains began to appear. The passively Q-switched laser possessed higher lasing threshold, which was caused by the energy loss of the insertion of ITO-NWAs. With the OC of T = 3%, the maximum average output power was 35 mW. To obtain higher output power, the OC of T = 3% was replaced with OC of T = 5%. The resonator reached a maximum output power of 58 mW, as shown in Figure 5. The main parameters for different transmissions as functions of the absorbed pump power are demonstrated in Figure 6. Under the absorbed pump power of 2.38 W with the OC of T = 5%, the corresponding shortest pulse duration, highest repetition rate and peak power were 490 ns, 17.09 kHz and 6.93 W, respectively. 

**Figure 7:**
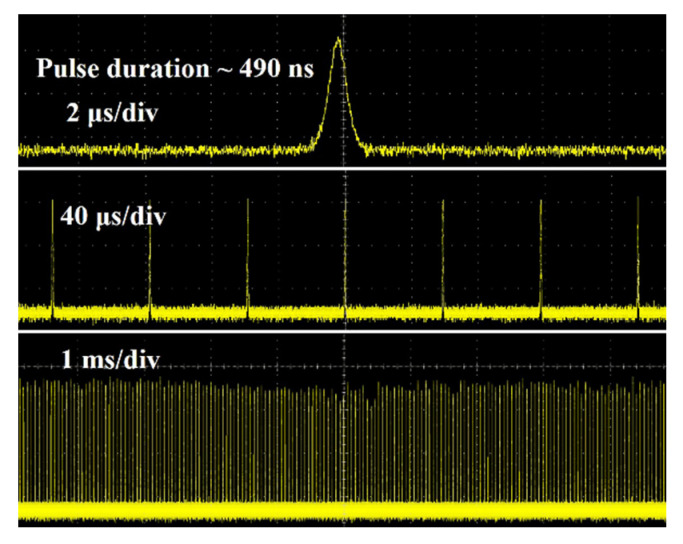
Typical Q-switched pulse trains at 2.8 μm when T = 5%.

Table 1 summarizes the comparison results of different OCs with transmittances of 3% and 5% for pulse lasers. The typical Q-switched pulse trains when T = 5% are shown in Figure 7. The sequence at 1 ms time scale demonstrated that the stability of the pulse laser performed well.

Table 2 summarizes the comparison results obtained from passively Q-switched performances at the 2.8 μm band. Through using different low-dimensional materials as SA, pulse lasers with their own characteristics were obtained. It should be pointed out that the experimental parameters obtained in this experiment are of high value compared with other work.

## 4. Conclusions

In summary, ITO-NWAs, as a form of nanomaterials, were manufactured by CVD method. Besides, prominent optical nonlinearity of ITO-NWAs was characterized at 2.8 μm. When ITO-NWAs were employed as a SA, stable pulse trains were successfully obtained in Er:Ca_0.8_Sr_0.2_F_2_ laser. Under the absorbed pump power of 2.38 W, passively Q-switched pulse laser with a 17.09 kHz repetition rate was carried out at the minimum duration of 490 ns, corresponding to the peak power of 6.93 W. It was the first time that ITO-NWAs were served as a SA at 2.8 μm in all-solid-state lasers, to the best of our knowledge. The experimental data manifested that ITO-NWAs are a promising SA in the MIR region.

## Figures and Tables

**Table 1:** Performances of the passively Q-switched laser under different OCs.

Transmittance of the OC	Output Power/mW	Shortest Pulse Width/ns	Repetition Rate/kHz	Peak Power/W	Single Pulse Energy/μJ
T = 3%	35	620	3.07	18.41	11.41
T = 5%	58	490	17.09	6.93	3.4

**Table 2:** Comparison of passively Q-switched laser performances at 2.8 μm.

Gain Medium	SA	Shortest Pulse Width/ns	Peak Power/W	Maximum Pulse Energy/μJ	Year
Er:ZBLAN	Black phosphorus	1180	/	7.7	2015 [44]
Er:Lu_2_O_3_	MoS_2_	335	23.8	8.5	2016 [45]
Er:Y_2_O_3_	Black phosphorus	4470	0.11	0.48	2016 [46]
Er:CaF_2_	Graphene	1324	2.07	2.74	2016 [7]
Er:YSGG	Bi_2_Te_3_/graphene	243	5.14	1.25	2017 [47]
Er:SrF_2_	Bismuth nanosheets	980	4.1	4.02	2018 [48]
Er:Y_2_O_3_	Graphene	296	8.77	2.59	2018 [49]
Er:CaF_2_	Graphene	632.9	5.85	3.7	2020 [16]
Er:CaF_2_	Bismuth nanosheets	607	5.35	3.25	2020 [50]
Er:Lu_2_O_3_	MXene Nb_2_CT_x_	223.7	16.96	3.79	2021 [51]
Er:Ca_0.8_Sr_0.2_F_2_	ITO-NWAs	620	18.41	11.41 (T = 3%)	This work
		490	6.93	3.4 (T = 5%)	
